# Transcriptional profiling of inductive mesenchyme to identify molecules involved in prostate development and disease

**DOI:** 10.1186/gb-2007-8-10-r213

**Published:** 2007-10-08

**Authors:** Griet Vanpoucke, Brigid Orr, O Cathal Grace, Ray Chan, George R Ashley, Karin Williams, Omar E Franco, Simon W Hayward, Axel A Thomson

**Affiliations:** 1 MRC Human Reproductive Sciences Unit, The Queens Medical Research Institute, Little France Crescent, Edinburgh EH16 4TJ, UK; 2 Departments of Urologic Surgery and Cancer Biology, Vanderbilt University Medical Center, 21st Avenue South, Nashville, TN 37232-2765, USA

## Abstract

**Background:**

The mesenchymal compartment plays a key role in organogenesis, and cells within the mesenchyme/stroma are a source of potent molecules that control epithelia during development and tumorigenesis. We used serial analysis of gene expression (SAGE) to profile a key subset of prostatic mesenchyme that regulates prostate development and is enriched for growth-regulatory molecules.

**Results:**

SAGE libraries were constructed from prostatic inductive mesenchyme and from the complete prostatic rudiment (including inductive mesenchyme, epithelium, and smooth muscle). By comparing these two SAGE libraries, we generated a list of 219 transcripts that were enriched or specific to inductive mesenchyme and that may act as mesenchymal regulators of organogenesis and tumorigenesis. We identified *Scube1 *as enriched in inductive mesenchyme from the list of 219 transcripts; also, quantitative RT-PCR and whole-mount *in situ *hybridization revealed *Scube1 *to exhibit a highly restricted expression pattern. The expression of *Scube1 *in a subset of mesenchymal cells suggests a role in prostatic induction and branching morphogenesis. Additionally, *Scube1 *transcripts were expressed in prostate cancer stromal cells, and were less abundant in cancer associated fibroblasts relative to matched normal prostate fibroblasts.

**Conclusion:**

The use of a precisely defined subset of cells and a back-comparison approach allowed us to identify rare mRNAs that could be overlooked using other approaches. We propose that *Scube1 *encodes a novel stromal molecule that is involved in prostate development and tumorigenesis.

## Abstract 2

Comparison of SAGE libraries for prostatic inductive mesenchyme and the complete prostatic rudiment revealed 219 transcripts that were enriched in, or specific to, inductive mesenchyme. Further analysis suggested that *Scube1 *is a novel stromal molecule involved in prostate development and tumorigenesis.

## Background

The mesenchymal compartment is involved in the induction and organogenesis of various organs, including lung, limb, kidney, pancreas, prostate, and mammary gland. In general, the process of organ induction begins with the formation of a specialized area of mesenchyme that acts upon adjacent epithelia to specify organ identity and subsequently dictates epithelial morphogenesis into the required form and function within the organ. The role played by inductive mesenchyme has been established using classical embryologic methods such as tissue recombination and engraftment, which have assayed the ability of spatially defined areas of mesenchyme to control morphogenesis and organogenesis. During organogenesis reciprocal interactions and signaling occur between the mesenchymal and epithelial compartments; in addition, numerous paracrine and autocrine growth regulatory pathways such as Wnt, hedgehog, fibroblast growth factor (FGF), Notch, and transforming growth factor-β are also active. The inductive mesenchyme involved in organ induction goes on to form signaling centers that are involved in growth and differentiation as well as specialized functions such as branching morphogenesis. At present, our knowledge of the pathways that are active in inductive mesenchyme is limited; this may be because of the inherently small size of these mesenchyma and a lack of suitable markers. It is likely that the proportion of inductive or specialized mesenchyme within a developing organ is low, which will make it difficult to isolate sufficient material for profiling studies.

The prostate develops from the embryonic urogenital sinus in response to testicular androgens and as a result of reciprocal mesenchymal epithelial interactions (for review [1]). Paracrine signaling from the urogenital mesenchyme (UGM) to the epithelium specifies prostatic epithelial identity, induces epithelial bud formation and growth, and regulates ductal branching morphogenesis (for review [2]). Androgen action within the urogenital sinus mesenchyme was originally defined as being necessary and sufficient for prostate organogenesis; androgen action in the epithelia is not required [3,4]. Within the mesenchyme a distinct area of mesenchyme has been defined that regulates prostatic organogenesis [5]. This mesenchyme has been termed the ventral mesenchymal pad (VMP), based on its anatomic position. However, it appears that the VMP is part of a structure that encircles the urethra and may participate in the formation of all lobes of the prostate. Additionally, it appears that the VMP is better anatomically defined in rat than in mouse, although it can be distinguished by its restricted expression of molecules such as FGF10 and bone morphogenetic protein (BMP)4 [6,7]. The VMP is present in both males and females, suggesting that androgens are not required for its formation [5,8], although androgens are required for prostate induction and organogenesis. The activity of molecules produced in the VMP may be indirectly regulated by androgens that control the formation of a layer of smooth muscle that is juxtaposed between the VMP and urethral epithelium [8,9]. The VMP constitutively expresses key growth regulatory molecules such as FGF10, which functions as a mesenchymal paracrine regulator of prostatic epithelia and is essential for the formation of the prostate [10].

Androgens are required for the formation of the prostate, and there has been considerable interest in defining the pathways that might be involved in mediating the effects of androgens. Furthermore, because androgen receptor activity is required in the mesenchyme/stroma, this has led to the idea that androgens may act through paracrine factors produced in the mesenchyme. At present there are no molecules that are expressed in the mesenchyme which show clear upregulation by androgens, despite a range of experimental approaches. It is also possible that androgens may not directly control the expression of paracrine acting factors but may act indirectly, by controlling the interaction of inductive mesenchmye with epithelia via the smooth muscle compartment. In the developing reproductive tract there is a sexually dimorphic layer of smooth muscle that separates inductive prostatic mesenchyme (VMP) from the urethral epithelium (from which nascent prostatic buds will form). In females this layer forms rapidly and isolates the VMP, but in males the layer remains discontinuous to permit interaction of the VMP with epithelia [8]. It appears that the smooth muscle patterning is controlled by androgens and estrogens [9]. The hypothesis that androgens act via the smooth muscle compartment would suggest that androgens may not directly regulate the expression of paracrine factors in the mesenchyme. This is supported by the observation that factors such as FGF10, which are required for the formation of the prostate, are equally abundant in males and females and do not appear to be regulated by testosterone [7,11].

The question of which genes are involved in androgen-driven growth of the prostate has led to several studies that have used arrays to examine the gene expression profile of the prostate and prostate cell lines. Such studies have used either whole prostate [12-15], prostate tumor samples [16-19], or prostate cell lines [20]. There is limited similarity between these datasets, which probably reflects the different nature of the tissues as well as the cellular heterogeneity in some of the tissues and samples. It may be hoped that a few genes were common to all studies, or within individual studies, that might identify mediators of androgen action upon growth. However, few or none appear to exhibit such a pattern. Additionally, it may be that only a subset of cells are the target of androgen action, in which case the identification of the gene expression signature of these cells within a complex tissue may be difficult [21]. This will be particularly difficult for low-abundance transcripts expressed in subsets of cells and in rare cells such as progenitor/stem cells.

It has recently become apparent that the stroma is also actively involved in neoplastic prostate growth (for review [22]). Tumor-associated stroma or reactive stroma exhibits a variety of phenotypic and functional differences relative to normal stroma (for review [23,24]). The tumor stroma is no longer able to restrain prostatic epithelial proliferation, but instead carcinoma-associated fibroblasts stimulate epithelial tumor growth [25,26] and stimulate tumor angiogenesis [27]. The role of stroma in prostate tumor growth is highly reminiscent of the developmental growth of the prostate, and developmental pathways have been identified in prostate tumor stromal cells [28]. This notion of developmental pathway involvement in tumorigenesis was pioneered by Pierce several years ago [29].

We have used an unbiased approach to identify new stromal regulators of prostate growth. Our thesis was that mesenchymal factors that are involved in prostatic induction would be constitutively expressed in either males or females, as predicted by the 'smooth muscle' hypothesis described above. Additionally, we speculated that our approach might identify potential 'andromedin' molecules if they were expressed at low levels in females, because females are exposed to low (nonmasculinizing) levels of androgens *in vivo*. Similarly, we thought that a highly sensitive approach would identify androgen regulated molecules at their un-induced levels in the female prostatic rudiment.

Using serial analysis of gene expression (SAGE) we profiled a subset of urogenital mesenchymal cells that comprise the VMP [30]. The VMP is a homogenous subset of mesenchymal cells that initiates and regulates prostate organogenesis, and which can be microdissected in sufficient quantity for SAGE library construction. In addition, we constructed a SAGE library of the whole prostatic precursor, comprising the VMP, smooth muscle and urethral epithelium (VSU). By comparing the two SAGE libraries, we hoped to identify molecules enriched or restricted to the VMP while eliminating those expressed throughout all tissues (such as housekeeping genes and genes expressed in smooth muscle and epithelium). Our SAGE library comparison yielded a list of 219 transcripts, which exhibited a statistically significant enrichment in the VMP compared with the whole precursor (VSU). Most of the 219 transcripts were identified by low frequency tags in the SAGE libraries, suggesting that they were derived from low-abundance transcripts. One of the molecules we identified was *Scube1 *[31]. We demonstrate that *Scube1 *is expressed during prostate induction and branching morphogenesis, and that it is restricted to a subset of prostatic mesenchyme including the VMP. Expression of *Scube1 *was not affected by androgens and was observed in both males and females. Additionally, *Scube1 *mRNA was expressed in prostate cancer stromal cells, and was downregulated in cancer-associated fibroblasts relative to normal prostate fibroblasts. We propose that the list of 219 transcripts that we have identified may contain several mesenchymal factors that are important during organogenesis and tumorigenesis.

## Results

### SAGE analysis of prostatic inductive mesenchyme

**Figure 1:**
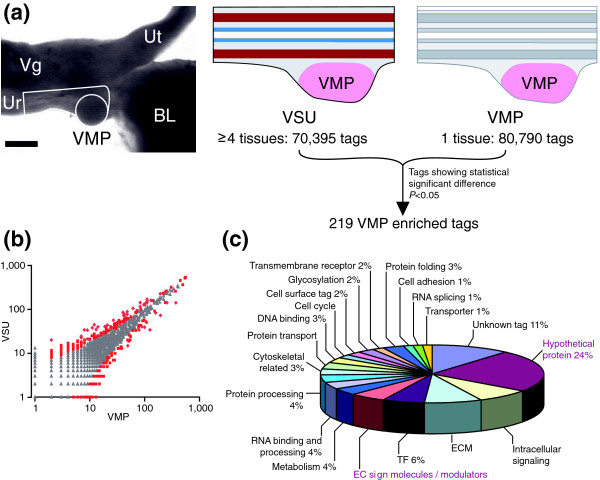
SAGE analysis of prostatic inductive mesenchyme. **(a) **The strategy used in construction and comparison of serial analysis of gene expression (SAGE) libraries to identify ventral mesenchymal pad (VMP) specific or enriched transcripts. P0 female urogenital tracts (UGTs) were microdissected to provide either pure VMP or the whole prostatic rudiment (VMP, smooth muscle, urethral epithelium [VSU]); the tissues dissected for library construction are outlined in black. The VMP tissue comprised only the condensed inductive mesenchyme of the VMP, whereas the VSU library contained urethral epithelium, smooth muscle, urethral mesenchyme and VMP. Both VMP and VSU SAGE libraries were sequenced to the indicated total number of tags. Pair-wise comparison was performed and 219 tags exhibiting statistically significant enrichment in the VMP were identified. **(b) **Scatter plot showing the comparison of the VMP and VSU SAGE libraries. Tag frequencies were plotted on a logarithmic scale and *P *values were calculated using the Z test; tags showing a difference at *P *= 0.05 are shown in red. **(c) **Pie chart depicting functional classification of the 219 VMP identified transcripts; extracellular (EC) signaling modulators and hypothetical proteins were highlighted for further analysis. Bl, bladder; TF, transcription factors; Ur, urethra; Ut, uterus; Vg, vagina; VMP, ventral mesenchymal pad.

To determine the transcript profile of prostatic inductive mesenchyme, we applied SAGE to a subset of the prostatic mesenchyme, namely the VMP. We constructed two SAGE libraries (Figure 1): one consisted purely of the VMP, whereas the second library (VSU) was composed of the whole prostatic precursor tissue containing VMP, smooth muscle, urethral epithelium and mesenchyme.

The area of the urogenital tract (UGT) dissected for library construction is shown in Figure 1a; the VMP can be seen as a sub-area of the VSU and both are outlined to illustrate the starting material for the libraries. The VSU library is made from a more complex tissue than the VMP library, and as a consequence, if both libraries are sequenced to a similar depth, VMP-specific transcripts will exhibit greater abundance in the VMP-only library. Our hypothesis was that transcripts expressed in the VMP would be 'diluted' in the VSU library (because the VMP is included as a component of the VSU). This effect would be most pronounced with regard to transcripts that were present at low abundance in the VMP and very low or absent in the VSU library. By comparing the VSU and VMP libraries and selecting for the tags with a significantly higher tag count in the VMP library (Figure 1a), we enriched for low abundance VMP-specific transcripts, while removing most housekeeping genes and broadly expressed transcripts. The number and frequency of tags showing a statistically significant difference between the two libraries is shown in Figure 1b. Datapoints colored red nearest to the VMP axis represent VMP-enriched tags, and these are described further below.

We sequenced about 70,000 tags for each library, translating into 22,755 and 26,932 distinct tags for the VSU and VMP libraries, respectively. About 68% of tags were found only once in each SAGE library. A significant proportion of these single tags will have resulted from sequence errors, and thus we excluded them from most subsequent analyses. Analysis of the VMP SAGE data revealed the presence of known mesenchymal regulatory factors in prostate growth, such as FGF10, BMP4, Smoothened and androgen receptor (AR), indicating an adequate sequencing depth to identify known regulators of prostate organogenesis.

### VMP-VSU SAGE library comparison to identify inductive mesenchyme specific transcripts

Comparison of the VMP and VSU SAGE libraries yielded a list of 219 tags that exhibited a significant enrichment in the VMP library (see Additional data file 1). SAGEMAP and genomic basic local alignment search tool (BLAST) were used to assign SAGE tags to specific transcripts and genes. The smallest statistically significant difference between our two SAGE libraries was 5:0 tags in VMP:VSU libraries [32]. Because an important goal of our studies was to identify mesenchymal paracrine factors, we examined our libraries for the presence of factors known to play a role in prostate development. We detected FGF10 in our VMP library, but it had four tag counts. As a consequence FGF10 was not identified as being VMP enriched in our SAGE screen, which exemplifies a limitation of our bioinformatic comparison. Although our approach identified many low abundance VMP enriched molecules, it inevitably will have missed some because of sampling error, and rare transcripts are most susceptible to sampling error. This is supported by estimation of the statistical power to determine differential expression between the VMP and VSU libraries, which indicates that at power of 0.9 differences of greater than twofold in tags of 50 and above might be detected [33,34]. The majority of transcripts in the VMP list are below this level, and it is likely that we have not identified all of the low abundance transcripts specific to the VMP. Although our analysis identified novel mesenchymal molecules that are involved in prostate organogenesis, it is possible that further such molecules remain to be identified.

The list of 219 VMP enriched tags/transcripts was functionally classified according to their Gene Ontology (Figure 1c). For approximately 11% of our list, we were unable to assign transcripts to these tags, but a number of them could represent anti-sense transcripts because they map to the 3'-untranslated region of known transcripts but in the anti-sense orientation (Additional data file 1; anti-sense tags are identified within the 219 list). We chose to focus on transcripts that encode potential growth regulatory molecules or modulators. About 5% of our list is made up of extracellular signaling molecules, whereas four tags mapped to known transmembrane receptors. We analyzed the large group of hypothetical proteins (25%) for the presence of signal peptides, transmembrane domains, and functional domains that suggested involvement in cellular signaling activity. As a result we identified a list of 17 putative extracellular or transmembrane signaling molecules that exhibited a significant enrichment in the VMP SAGE library (Table 1).

**Table 1:** Putative secreted or cell surface signaling molecules that show significant enrichment in the VMP SAGE library

LONG-SAGE tag	VMP	VSU	Uni-gene	Description (SAGEMAP)	Genomic BLAST
CATTTTCTGGCAAAATC	124	34	964	Insulin-like growth factor 2	
CCTAGCCCCTCCCACCA	49	15	7961	*Rattus norvegicus *similar to latent transforming growth factor-β binding protein 4S (LOC292734), mRNA	
ATATAATGAATAATAAT	38	13	14547	Delta-like homolog (*Drosophila*)	
GTTTGTACAATAAATAC	14	4	37338	Latent transforming growth factor-β binding protein 3	
GATGAATGTTATATGTT	12	2			Unique hit, 2 kb from mRIKEN cDNA1200009O22
TGAATCCTCTCCCTAAA	11	2	15332	*R. norvegicus *similar to RIKEN cDNA 9430096L06 (LOC291813), mRNA	Unique hit, close to novel transcript (h Chemokine like superfamily factor 3)
TAAAGTCAAAATAAAAT	11	1	8257	*R. norvegicus *transcribed sequences	Unique hit, 2 kb from Semaphorin6D locus
TGGGCATAGCTGAGGTG	10	2	41133	*R. norvegicus *transcribed sequences	Unique hit, 2 kb from novel transcript with similarity to mSorC2 precursor (VPS10 domain containing receptor
TAAGAGCTCTTTCCATC	10	1	8672	*R. norvegicus *similar to hypothetical protein, estradiol-induced (LOC308843), mRNA	Unique hit, ortholog of chicken Tsukushi
TCTGAATATAACATATC	8	1	22787	*R. norvegicus *similar to sprouty 1 (LOC294981), mRNA	
CCGCTTGAGACTCCTTC	6	0	25124	Rat insulin-like growth factor I mRNA, 3' end of mRNA	
GCATAGTCTGAGATGCA	6	0	40510	*R. norvegicus *transcribed sequences	Unique hit, 2 kb from Wnt4 locus
TTCCTGACTAAATGTAG	6	0	65930	Notch gene homolog 2 (*Drosophila*)	
CCTTGGGGGAGGGTGGG	5	0			Unique hit, 1 kb from to mSlit2 homolog
GGAGATACCTGTTCAAA	5	0	11567	Nel-like 2 homolog (chicken)	
TAATTAAACACTTGTGA	5	0	103231	*R. norvegicus *transcribed sequences	Unique hit, 4 kb from novel transcript with homology to m*Scube1*
AGTGTGTACAAGCTTAG	5	0			Unique hit, close to novel transcript similar to mEphB3 receptor

BLAST, basic local alignment search tool; kb, kilobases; SAGE, serial analysis of gene expression; VMP, ventral mesenchymal pad; VSU, VMP, smooth muscle and urethral epithelium.

We examined several members of the VMP-enriched list by quantitative RT-PCR, Northern blot, and whole-mount *in situ *hybridization to determine whether they could be verified as VMP enriched. The candidates that we chose to examine further included secreted or membrane-bound molecules that might be involved in cell-cell interactions. Of 30 candidates tested, 11 were confirmed as being VMP enriched, 12 were not confirmed, and seven were inconclusive. The transcripts that were validated as VMP enriched were as follows: Igf2, MMP2, Dlk1, Notch 2, Nel-like2, decorin, EphB3 receptor, slit2, sprouty1, mSorC2m, and sema6D. These candidates comprise 11 of the 17 extracellular or transmembrane molecules listed in Table 1. We estimate that approximately one-third of the transcripts in the VMP list may be confirmed as VMP specific by additional follow up, but this will require further experimental validation. Members of the insulin-like growth factor family have been implicated in prostate organogenesis [35,36]. Also, Wnt4 was recently reported to be expressed in developing prostates, but its precise localization was not determined [37]. Our SAGE data revealed the expression of a number of transcription factors that have been implicated in organogenesis of other tissues (for example, PLAG1, Pbx3, and SOX7). In addition to intracellular molecules, there was a considerable number of extracellular matrix proteins. The VMP consists of mesenchyme that is morphologically distinct from the surrounding mesenchyme. The higher expression of some of these extracellular matrix components may be responsible for the different morphology of the inductive mesenchyme. In general, our VMP-enriched list gives an overview of the transcriptional programs that are active in mesenchyme during prostate organ induction.

### *Scube1*: a new prostate inductive mesenchyme specific gene

**Figure 2:**
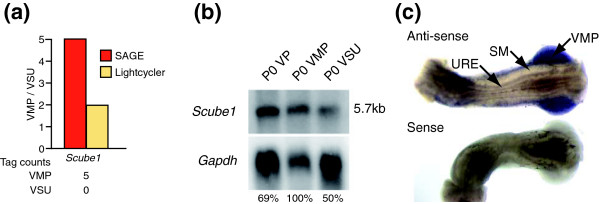
Localization of *Scube1 *to the inductive mesenchyme (VMP) of female UGT. **(a) **Comparison of *Scube1 *transcript levels in ventral mesenchymal pad (VMP) and VSU (VMP, smooth muscle and urethral epithelium) using serial analysis of gene expression (SAGE) and quantitative RT-PCR. Red bars represent the SAGE data, and yellow bars represent the quantitative RT-PCR data (normalized to TBP levels). *Scube1 *mRNA was found to be enriched in the VMP by both SAGE and quantitative RT-PCR analyses. **(b) **Northern analysis showing a twofold enrichment of *Scube1 *mRNA levels in the VMP compared with the VSU. **(c) **RNA whole-mount *in situ *hybridization of *Scube1 *in P0 female UGT; anti-sense probe is at top of panel and sense is at the bottom of the panel. *Scube1 *transcripts localized to the VMP, and were not observed in smooth muscle (SM) and urethral epithelium (URE).

**Figure 3:**
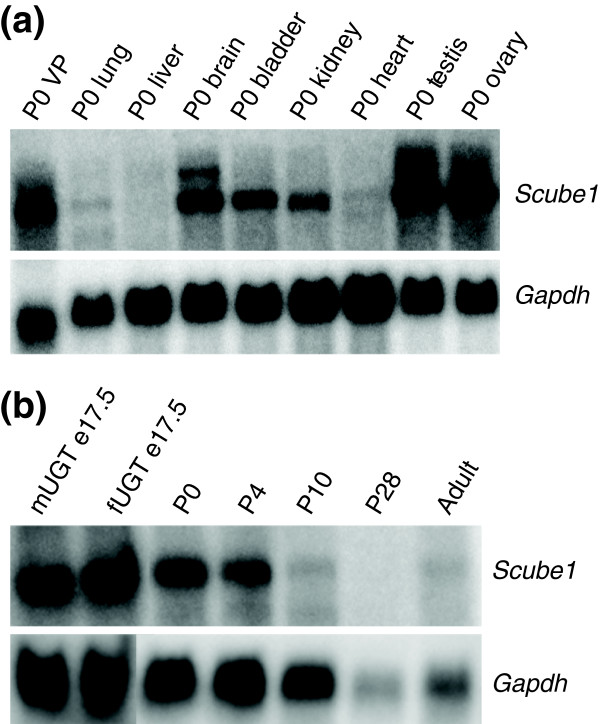
Expression of *Scube1 *mRNA in rat P0 tissues and during prostate development. **(a) **Northern blot analysis of *Scube1 *mRNA levels in P0 tissues; highest levels of *Scube1 *transcripts were observed in testis and ovary. Brain, ventral prostate (VP), bladder, and kidney showed moderate *Scube1 *expression. Kidney and lung showed very low expression, and liver was negative. **(b) **Expression of *Scube1 *in male and female urogenital tract (UGT) at E17.5, and subsequent expression the VP at P0, P4, P10, P28, and adult.

One of the extracellular signaling molecules identified as being VMP enriched by SAGE analysis was *Scube1 *[31]. Five *Scube1 *tags were present in the VMP library, whereas none were identified in the VSU library (Figure 2a). The enrichment of *Scube1 *in VMP RNA was confirmed by both quantitative RT-PCR (Figure 2a; yellow bar) and Northern blot (Figure 2b). *Scube1 *mRNA was also identified in P0 prostate (Figure 2b). Whole-mount RNA *in situ *hybridization further defined expression of the *Scube1 *transcript only in the mesenchyme of a P0 female UGT, whereas the peri-urethral mesenchyme and the urethral epithelium did not express *Scube1 *(Figure 2c). Levels of *Scube1 *expression in the UGT, prostate, and inductive mesenchyme have not previously been reported. Grimmond and coworkers [31] isolated the *Scube1 *transcript from a cDNA library of the mouse urogenital ridge and reported expression in developing gonads, nervous system, and mesenchyme of developing limb buds. Our Northern blot analysis in P0 tissues identified the highest *Scube1 *expression in testis and ovary, followed by high expression in prostate and brain (Figure 3a). We could barely detect *Scube1 *in adult tissues (Figure 3b and data not shown). It must be noted that there are significant developmental differences in the organs at P0; the prostate is rudimentary and undergoing extensive branching morphogenesis, whereas organs such as lung and kidney are more mature. The expression pattern in rat P0 tissues is somewhat different than the reported expression pattern in adult human tissues, which may be due to different developmental stages of organ development [38]. The decrease in *Scube1 *transcript levels between embryonic and adult stages may be a result of either gene downregulation or loss of the subset of cells that express it, and we cannot be sure which is the primary factor or whether the decrease is a result of both downregulation and loss of cells.

To further examine *Scube1 *expression in the prostate, we compared *Scube1 *transcript levels in early UGTs, developing prostate undergoing branching morphogenesis, and mature adult prostate (Figure 3b). *Scube1 *mRNA levels were most abundant during prostate induction at E17.5 (before bud development), and were high during prostate branching and growth (P0 and P4). By P10 there was significant decrease in *Scube1 *mRNA levels, with very low or undetectable levels by puberty (P28) and in the adult rat. This temporal distribution suggested a role for *Scube1 *in prostate organogenesis. However, we observed similar levels of *Scube1 *mRNA in both males and females at E17.5, which suggested that there was no sexually dimorphic difference in *Scube1 *transcript expression. The *Scube1 *mRNA encodes a secreted glycoprotein with epidermal growth factor repeats and a CUB domain (a domain first found in complement C1r, C1s, uEGF, and bone morphogenetic protein 1). No function has yet been described for mammalian *Scube1*, but its domain structure suggests a possible role in growth factor modulation [31]. Studies in zebrafish have suggested that Scube family members may be involved in sonic hedgehog (Shh) signal transduction, and it is possible that Scube may control other extracellular signaling pathways [39,40].

### Spatial localization of *Scube1 *mRNA during prostate development

**Figure 4:**
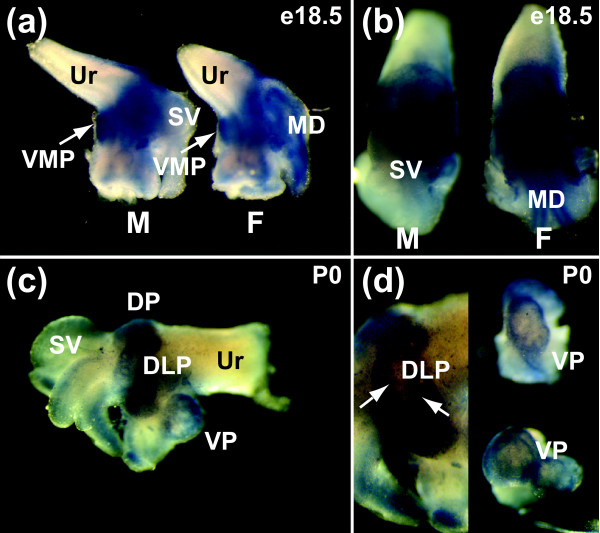
Spatial distribution of *Scube1 *mRNA in male and female UGT. **(a,b) **Whole-mount *in situ *hybridization showing *Scube1 *transcript expression in E18.5 male (M) and female (F) urogenital tract (panel a shows lateral view and panel b shows dorsal view). *Scube1 *mRNA was present in a subset of the urogenital mesenchyme including the VMP (marked by arrows in male and female). In females there was staining in the mesenchyme of the Mullerian duct (MD). In males, the seminal vesicle (SV) mesenchyme showed little or no staining. In both sexes the urethra (Ur) and urethral mesenchyme were negative for *Scube1 *mRNA. **(c,d) **P0 male urogential tract. The mesenchyme of the dorsal prostate (DP), dorsolateral prostate (DLP), and ventral prostate (VP) showed *Scube1 *transcript expression. In panel d, DLP is shown on the left hand side and epithelial buds (arrows; negative for *Scube1*) can be seen entering the DLP mesenchyme. On the right hand side of panel d ventral prostate is shown, and *Scube1 *transcripts are abundant in the mesenchyme and show enrichment in the peripheral mesenchyme.

Some insight into the role played by *Scube1 *in prostate growth was obtained by defining the cell and tissue compartment expression pattern. Whole-mount *in situ *hybridization was used to determine the spatial expression pattern of *Scube1 *at different stages of prostate development. There was robust *Scube1 *mRNA expression in the urogenital sinus (UGS) during early prostate organogenesis in rat, at fetal day E17.5 (Figure 3b). Shortly after this time point prostatic budding is initiated, when developing epithelial buds penetrate into the surrounding UGM in the dorsal, lateral, and ventral directions. In E18.5 UGTs, *Scube1 *mRNA was present in the VMP of both males and females (Figure 4a,b), and the patterns of *Scube1 *expression around the urethra of male and female were very similar. In males, *Scube1 *transcripts were present in the mesenchyme overlying the position where dorsal and lateral prostates formed (Figure 4a,b), whereas in females it was also present in the Mullerian duct. The developing seminal vesicles and Wolffian duct structures of male E18.5 UGTs exhibited very little or no *Scube1 *expression (Figure 4b). In P0 male UGTs a similar expression pattern was seen; robust *Scube1 *mRNA levels were observed in the mesenchyme, whereas the emerging prostatic epithelial ducts were negative for *Scube1 *(Figure 4c,d). In the dorsolateral prostate the *Scube1 *signal was strongest in the mesenchyme directly adjacent to the ducts (Figure 4d). *Scube1 *transcripts were also observed in the mesenchyme of the ventral prostate (VP; Figure 4c,d). Taken together, it appeared that *Scube1 *was expressed in a specific subset of the mesenchyme, consistent with the VMP tissue used in the construction of the SAGE libraries.

### *Scube1 *expression is not regulated by testosterone

The spatiotemporal localization of *Scube1 *suggested that it might function as a regulator of prostate growth. To determine whether *Scube1 *expression was regulated by androgens, we examined whether *Scube1 *mRNA expression and localization were affected by testosterone using male or female UGT rudiments grown *in vitro*.

**Figure 5:**
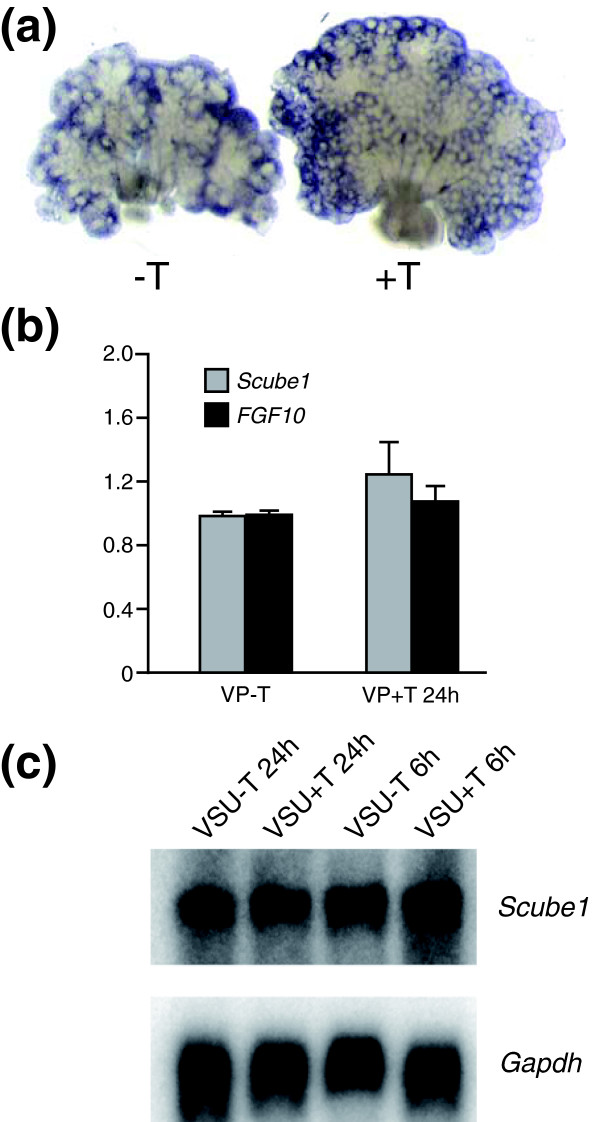
Testosterone does not alter *Scube1 *mRNA levels or expression pattern. **(a) **Whole-mount RNA *in situ *hybridization of ventral prostates (VPs) grown *in vitro *for 6 days in the absence (-T) or presence (+T) of testosterone. **(b) **Quantitative RT-PCR for *Scube1 *and *FGF10 *mRNAs in VPs grown *in vitro* with/without testosterone. VPs were cultured in the absence of testosterone for 3 days followed by an incubation of 24 hours in the presence or absence of testosterone. **(c) **Northern analysis for *Scube1 *mRNA on P0 female urogenital tracts treated *in vitro *with testosterone for 6 hours and 24 hours. VSU, ventral mesenchymal pad, smooth muscle, urethra.

Whole-mount RNA *in situ *hybridization of male VPs grown in the absence or presence of testosterone exhibited little or no change in transcript distribution; *Scube1 *localized to the inductive mesenchyme surrounding the distal duct tips under both conditions (Figure 5a) and was absent from epithelia. *Scube1 *mRNA showed slightly increased expression in the mesenchyme at the periphery of the organ, where epithelial proliferation is highest [41]. To quantify changes in *Scube1 *transcripts we grew VPs in the presence or absence of testosterone and measured transcript levels by quantitative RT-PCR (Figure 5b). Treatment of VPs with testosterone had no significant effect on *Scube1 *or FGF10 mRNAs. To rule out potential carry over of testosterone in cultures of male VPs, we used cultures of P0 female UGTs grown *in vitro*. The rudiments used correspond to the VSU used for SAGE library construction. Treatment of P0 female UGTs for 6 or 24 hours with testosterone did not change *Scube1 *transcript levels, as shown by Northern analysis (Figure 5c). In addition, we examined *Scube1 *mRNA levels in primary VMP mesenchymal cells grown *in vitro *[42], and no changes were observed following short-term or long-term treatment with testosterone (data not shown).

Taken together, it appears that androgens do not alter the distribution of *Scube1 *mRNA in males, or the amount of *Scube1 *mRNA in either males or females. Furthermore, we did not observe a difference in *Scube1 *levels between E17.5 male and female embryonic UGTs *in vivo *(Figure 3b), and we conclude that *Scube1 *is unlikely to be regulated by androgens.

### *Scube1 *expression is downregulated in prostatic cancer-associated fibroblasts compared with normal prostate fibroblasts

Because *Scube1 *was specifically expressed in the mesenchyme during development, we examined whether it was present in prostate cancer stroma and whether it was differentially expressed between cancer-associated fibroblasts (CAFs) and normal prostate fibroblasts (NPFs). *Scube1 *mRNA was examined in five pairs of functionally tested NPF and CAF samples by both Northern analysis and quantitiative RT-CPR. All CAFs had been shown to produce tumors when recombined with an epithelial cell line, whereas all NPF samples did not [26] (and data not shown). Four pairs of CAFs/NPFs were matched from the same patient, whereas one pair was not.

**Figure 6:**
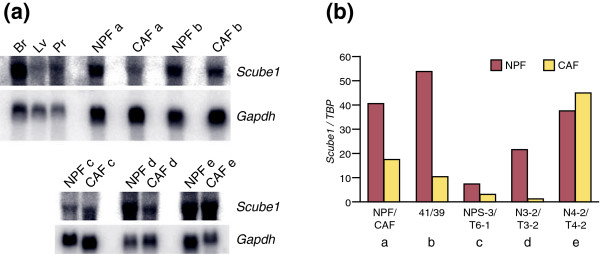
Expression of *Scube1 *mRNA in prostate tumor stromal cells using CAFs and NPFs. **(a) **Northern analysis of *Scube1 *mRNA in five pairs (a to e) of cancer-associated fibroblasts (CAFs)/normal prostate fibroblasts (NPFs). Embryonic human brain, liver, and prostate are included as control tissues, and RNA loading is illustrated by hybridization with *Gapdh*. *Scube1 *mRNA was lower in CAFs in four out of five CAF/NPF pairs. **(b) **The downregulation of *Scube1 *mRNA in CAFs was confirmed by quantitative RT-PCR; *Scube1 *mRNA levels were normalized to *TBP *mRNA levels. Br, brain; Lv, liver; Pr, prostate.

*Scube1 *transcripts were identified in all CAFs and NPFs, demonstrating that *Scube1 *was expressed in prostate cancer stromal cells (Figure 6). Furthermore, in four out of five samples *Scube1 *was found to be downregulated in the CAFs compared with the NPFs, by both Northern blotting and quantitative RT-PCR (Figure 6). *Scube1 *downregulation was between 2-fold and 20-fold. This decreased expression in CAFs compared with NPFs could have been caused by loss of a specific subset of cells in the CAF culture versus the NPF culture. However, because these cell populations are stable in culture and this effect is observed in different sets of patient matched NPFs/CAFs, we propose that the difference in expression between the cell populations is most likely caused by specific loss of *Scube1 *expression in CAFs either by downregulation or by loss of the chromosomal region. The same samples were also checked for CXC chemokine ligand (CXCL)12 mRNA levels; CXCL12 has been identified as a stromal molecule that stimulates tumorigenesis [43]. In four of five samples, CXCL12 was found to be upregulated in the CAFs (data not shown), similar to reported findings in breast tumor stroma [44].

## Discussion

In this study we provide a detailed molecular profile of a subset of the mesenchymal cell compartment, the VMP, which controls prostatic organ induction and development. The UGM/urogenital stroma is a very potent tissue during both development and disease, which has been demonstrated by tissue recombination experiments. Androgen action in the UGM has been shown to be necessary and sufficient for prostatic development (for review [1]). When recombined with human embryonic stem cells, the UGM directs differentiation into mature human prostate tissue expressing prostate-specific antigen [45]. Furthermore, embryonic UGM has the ability to re-differentiate prostate cancer cells and to reduce tumor growth [46]. It has recently emerged that the stroma can initiate and stimulate prostate tumorigenesis [25-27,47], and profiling of tumor stroma has identified developmental molecules such as secreted frizzled-related protein 2 [28]. Because of the restricted expression of *Scube1 *in a small subset of cells, it would be very difficult to identify *Scube1 *in a profiling screen of heterogeneous tissue samples such as tumors unless it was significantly upregulated during tumorigenesis. Hence, a transcript profile of a potent tissue such as the VMP not only provides us with potential new regulators of prostate growth, but it may also highlight some that could regulate neoplastic growth.

For our analysis we used the inductive mesenchyme of a female UGT and assumed that key prostatic regulators may not be induced by testosterone [48]. We also reasoned that a highly sensitive gene profiling approach might detect androgen-regulated molecules at their 'un-induced' levels, in the event that some stromal mediators might be upregulated by androgens. Most profiling studies have focused on pathways activated by androgens to find new regulators of prostate growth [12-15]. However, none of these studies has successfully identified molecules that satisfy the criteria of being 'andromedins'. At present no growth factors expressed in the UGM have been shown to be directly regulated by androgens. We hypothesized that key prostatic inducers are constitutively expressed in the inductive mesenchyme, regardless of testosterone levels, and that by profiling the VMP novel growth regulatory signaling pathways would be identified. We have previously suggested that molecules produced by, or in, the VMP may be indirectly regulated by an androgen sensitive layer of smooth muscle that forms a separating layer between the VMP and the urethral epithelia [8,9].

To identify VMP-specific transcripts from our VMP SAGE data, we employed a novel strategy. We compared the VMP-only SAGE library with a more complex SAGE library of the complete female prostatic precursor (termed VSU). By doing so we specifically focused on low abundance VMP-enriched or VMP-restricted transcripts. Additionally, the ability to isolate enough inductive mesenchyme for direct SAGE library construction (without amplification or dilution with other cell types) indicates that our VMP library may contain a number of important and potent molecules that are absent or poorly represented in current datasets, because these are typically made from tissues composed of many cell types. Profiling of the VMP, which is highly enriched for growth regulatory proteins such as FGFs, yielded a number of extracellular and transmembrane proteins with putative growth regulatory or modulatory functions. The expression of many of these factors in the prostate has not been revealed by other profiling studies, which may be because of their greater cellular complexity or the use of adult tissues in which growth regulatory pathways are less active. We estimate that 30% to 50% of the molecules in the VMP list will be experimentally confirmed as being VMP enriched, based on our follow up of 30 candidate molecules. This ratio compares favorably with other profiling studies, but it is inevitable that transcripts will have been missed and that others will not be experimentally confirmed. This is likely because our studies have focused upon low abundance transcripts, which are the most susceptible to sampling error when measured using SAGE.

We identified Scube1 as a prostatic inductive mesenchyme specific molecule. The temporal and spatial expression pattern of *Scube1 *during prostate organogenesis is coincident with prostate induction and subsequent branching morphogenesis. In developing ventral prostates the highest concentration of *Scube1 *transcript was localized to the mesenchymal cells adjacent to the distal duct tips. This localization to the distal mesenchyme mirrors the localization of *Fgf10 *and suggests an involvement of *Scube1 *in ductal growth. Interestingly the Shh receptor Ptc also localizes to this inductive mesenchyme [11]. Although no function has yet been described for *Scube1*, its family member *Scube2*, is reported to be involved in Shh signal transduction [39,40,49]. Studies in zebrafish highlighted *Scube2 *as an essential mediator of hedgehog (Hh) signaling with a role in stabilization or transport of the Hh protein, or a role in the endocytotic uptake of Shh [40,49]. During prostate development, Shh signaling regulates ductal growth and branching, although it is not essential for prostate induction [50,51]. Shh is composed of prostatic epithelium and acts as a mitogen for the prostatic mesenchyme. *Scube1 *expression in the target mesenchyme may be required for the mitogenic effects of Shh. Because several components of the Hh pathway are regulated by Shh, we examined whether *Scube1 *levels in P0 UGTs and VPs were affected by Shh treatment. We could not detect any regulation of *Scube1 *transcript expression by recombinant Shh or inhibition of Hh signaling with cyclopamine (data not shown). It is possible that Scube may regulate other signaling pathways because it is expressed in areas where Hh signaling is not thought to be important. Studies in zebrafish also suggested that Scube family members may modulate BMP activity [39]. To determine the function of SCUBE1 protein, we have attempted to purify recombinant *SCUBE1*, but in our studies it appeared that SCUBE1 became insoluble when purified and we were unable to assess the action of the protein in cell and organ culture studies. *Scube1 *has been detected in vascular endothelial cells, and the protein can form oligomers that are associated with the cell surface [38]. Gene targeting studies or mis-expression approaches will be needed to assess the role of *Scube1 *in prostate organogenesis.

We did not observe any regulation of *Scube1 *mRNA by androgens *in vivo *or *in vitro*, and therefore it is unlikely that *Scube1 *functions as an andromedin. Both *Fgf10 *and *Fgf7 *are important regulators of prostate growth and neither is androgen regulated *in vivo*. It is likely that there is a group of molecules important in prostate growth that are not regulated by androgens. *Scube1 *has not been identified in profiling studies looking for androgen regulated mediators of prostate growth, and it seems probable that *Scube1 *is not a direct mediator of androgen action. *Scube1 *specific tags are present in SAGE libraries made from mouse E16.5 UGM [37]. In the study conducted by Zhang and coworkers [37] the tag count for *Scube1 *was lower than that in our study, and *Scube1 *would not have been identified as inductive mesenchyme specific, because those authors did not profile subsets of the mesencyhmal compartment. Because of the restricted expression of *Scube1 *in a small subset of cells and the low abundance of this transcript, it would be very difficult to identify *Scube1 *in a profiling screen using whole prostate organs or a complex tissue such as tumors.

We showed that *Scube1 *is expressed in both prostate development and prostate cancer stromal cells, which concurs with the observation that many developmental pathways are involved in tumorigenesis. The downregulation of *Scube1 *in CAFs compared with NPFs suggests that it may function as a tumor suppressor, although this remains to be experimentally confirmed. *Scube1 *is located on human chromosome 22q13, and this region is reported to be deleted in some prostate cancer samples [52,53], which supports the notion that *Scube1 *may function as a tumor suppressor. The region 22q13 contains approximately 242 genes, and thus genes other than *Scube1 *may be acting as tumor suppressors. Also, it is not known whether the deletion of 22q13 is present in stroma or epithelia within the tumor samples. Although we do not know whether *Scube1 *is expressed in epithelia, there is no indication that it is expressed in epithelia during development and it appears to be absent from some prostate epithelial cell lines (Vanpoucke G, Thomson AA, unpublished data). *Scube1 *has not been observed in prostate cancer using whole tumor profiling studies [54,55], perhaps because it is expressed in a small subset of cells within the tumor that are not well represented in whole tumor gene signatures. The only way to identify molecules in small subsets of tumor will be to increase the efficiency of whole tumor profiles (by increasing the sampling level) or to isolate subsets of the tumors for profiling [44]. We have identified tumor expression of *Scube1 *using a candidate-based approach, based upon its expression in a subset of mesenchyme that is known to be important in prostate development.

Our study identified several additional signaling molecules that were expressed in the inductive mesenchyme, which have the potential to act either as paracrine regulators of the prostatic epithelium or as mediators of reciprocal epithelial-mesenchymal signaling during prostate organogenesis. Estradiol-induced gene 4 (E2IG4) was originally identified in a screen for estrogen responsive genes [56]. Furthermore, it is the mammalian ortholog of chicken Tsukushi, which is a BMP inhibitor that is involved in organizer induction [57]. Dlk-1 is known as a de-differentiation factor from studies of adipogenesis [58]. Its expression in prostate mesenchyme has not previously been reported, but it was observed in a SAGE screen for stromal changes in breast cancer [44]. Nell2 is a secreted epidermal growth factor family member; no function has been described for this protein, but it was shown to be over-expressed in benign prostatic hyperplasia [59]. Sprouty is a negative regulator of FGFs and its expression is reported to be decreased in prostate cancer [60]. Members of the Slit, Semaphorin, and Ephrin families of proteins are best known for their role as guidance cues for axons (for review [61]), but recent studies show that they contribute to the development of a variety of organs. Slit2 plays a key role during kidney development in positioning the site of kidney induction [62].

## Conclusion

We identified *Scube1 *as a novel prostatic inductive mesenchyme specific molecule with potential roles in prostate development and disease. Furthermore, our VMP-specific SAGE list gives an overview of the transcriptional programs active in a key subset of the mesenchyme during prostate induction. This detailed analysis of the developmental pathways that control normal prostate morphogenesis can also provide insights into the regulatory pathways that control neoplastic growth.

## Materials and methods

### RNA isolation and SAGE library construction

Whole prostatic precursors (VSUs) and VMPs were microdissected from female postnatal day 0 (P0) outbred Wistar rats. RNA was extracted from 100 pooled VSUs and 90 VMPs using the RNeasy Mini kit (Qiagen, Crawley, UK). RNA quality and concentration was measured using an Agilent (Santa Clara, CA, USA) 2100 Bioanalyser; 50 μg VSU total RNA and 20 μg of VMP total RNA were used. Long-SAGE libraries were constructed using the I-SAGE kit (Invitrogen, Carlsbad, CA, USA) protocol adapted for LongSAGE according to the Long-SAGE protocol (released by Genzyme, Cambridge, MA, USA, January 2003). *NlaIII *and *MmeI *were used as the anchoring and tagging restriction enzymes, respectively. Before ligation into the pZERO-1 vector, concatemers were digested with *SphI *to improve their cloning efficiency. The VMP and VSU SAGE libraries contained 80,790 and 70,395 tags (respectively) and are shown in Additional data files 2 and 3 (Gene Expression Omnibus accession number GSE7899).

### Bioinformatic analysis of SAGE data

Long-SAGE tags were extracted from the raw sequence files, filtered, and tabulated using SAGE2000 software (version 4.5) [63]. During this process linker sequences and duplicate dimers were removed from the sequence data. For the VMP and P0 UGT pair-wise library comparison, a tag was tested for significance using the Audic and Claverie [32,64] and the Z-test (Statview software package, Letchworth, UK). A 95% confidence interval was applied (*P *< 0.05). Both statistical tests gave similar results. Tag to gene annotations were done using the rat Long-SAGE map reference database [65]. When no gene was assigned with a tag, a genomic BLAST was performed with the complete 21 base pair tag sequence (CATG-17bp tag) using the ENSEMBL rat genome browser to aid in gene assignment [66]. When a tag gave a unique hit in the rat genome within 5 kilobases from an assigned locus, we hypothesized that the tag originated from this transcript. This manual tag mapping was verified for a number of transcripts by performing rapid amplifications of cDNA 3' ends (3'-RACE) experiments [67]. The assignment of molecular function of the genes was done using the Gene Ontology database and Genecards [68,69].

### Quantitative RT-PCR and Northern analyses

Quantitative RT-PCR and Northern analysis were performed to validate the SAGE data, using several independent isolates of VMP and VSU RNA. PCRs were performed on the Lightcycler using the Lightcycler FastStart DNA master SYBRGreen kit (Roche, Burgess Hill, UK). Primer pairs were designed to amplify the genes summarized in Table 2.

**Table 2:** Primer pairs used in the present study

Gene	Forward primer	Reverse primer
r*Scube1*	GTTCTCCAGGCTTCTTCTCAGAG	TACAGTGGGCAGAGCATTGG
r*FGF10*	CAGTGGAAATCGGAGTTGTTG	ATGACGCAATGACACAGAGCT
r*TBP*	GCGGTTTGGCTAGGTTTCTG	CCTAGAGCATCCTCTTATCTCCT
h*SCUBE1*	ATTTGTAGGGCCGCAGGAAC	CGAGTCTGGCACGAAGAGTG
h*Gapdh*	TTAGCACCCCTGGCCAAGG	CTTACTCCTTGGAGGCCATG

The amplified PCR products were used as template for DNA probe synthesis for Northern hybridization. DNA probes were labeled using the Radprime DNA labeling kit (Invitrogen) in the presence of α^32^P dCTP. Transcript abundances were normalized to either *TBP *or *Gapdh *housekeeping gene.

### Whole-mount RNA *in situ *hybridization

A 739 base pair fragment corresponding to nucleotides 2,258 to 2,996 from r*Scube1 *(XM_235529) was amplified and cloned into the pCR4-TOPO vector (Invitrogen). Sense and anti-sense probes were transcribed and labeled with digoxigenin using T7 and T3 RNA polymerase. Dissected tissues were fixed in 4% paraformaldehyde at 4°C overnight, dehydrated through graded methanol, and stored in 100% methanol at -20°C. RNA *in situ *hybridization on embryonic and P0 UGTs and cultured ventral prostates were performed using the InsituPro VS robot (Intavis, Bioanalytical Instruments AG, Cologne, Germany). After rehydration tissues were bleached in 6% hydrogen peroxide, treated with 20 μg/ml proteinase K for up to 1 hour and refixed in 4% paraformaldehyde/0.2% glutaraldehyde. Hybridization with digoxigenin-labeled probes was performed at 65°C for 16 hours, followed by high stringency washes. For detection tissues were incubated with anti-digoxigenin antibody conjugated to alkaline phosphatase (Roche, Burgess Hill, UK; 1/2,000) at 18°C for 6 hours. After washing, the color was developed using NBT/BCIP solution. Typical development times were around 8 hours.

### Organ culture and *in vitro *testosterone treatments

P0 VPs were cultured for 6 days in presence or absence of 10^-8 ^mol/l testosterone, as previously described [7], before whole-mount *in situ *hybridization. Additionally, P0 VPs were cultured in the absence of testosterone for 3 days, followed by 24 hours of treatment with testosterone and subsequent RNA preparation. P0 female UGTs were cultured under similar conditions and treated with testosterone for 6 or 24 hours, followed by RNA isolation.

### Culture of CAFs and NPFs

Cells were isolated and grown as described by Olumi and coworkers [26]. Prostate tissue was cut into 1 to 2 mm^3 ^pieces and treated with collagenase type I (225 units/ml; Sigma) and hyaluronidase (125 units/ml; Sigma) in RPMI 1640 (with 10% fetal calf serum) overnight at 37°C. Cells were washed twice with media, plated, and grown until approximately 50% confluence. Stromal cells were selected using differential trypsinization, with 0.05% Trypsin, to remove only the stromal cells, which were subsequently passaged two or three times. Immunohistochemistry for vimentin and smooth muscle α-actin were used as stromal markers, and epithelial contamination was excluded using pan-cytokeratin staining. CAF and NPF cells were recombined with BPH1 cells to determine tumorigenic activity [26].

## Abbreviations

BLAST, basic local alignment search tool; BMP, bone morphogenetic protein; CAF, cancer-associated fibroblast; CXCL, CXC chemokine ligand; FGF, fibroblast growth factor; Hh, hedgehog; NPF, normal prostate fibroblast; RT-PCR, reverse transcription polymerase chain reaction; SAGE, serial analysis of gene expression; Shh, sonic hedgehog; UGM, urogenital mesenchyme; UGS, urogenital sinus; UGT, urogenital tract; VMP, ventral mesenchymal pad; VP, ventral prostate; VSU, VMP, smooth muscle and urethral epithelium.

## Authors' contributions

GV designed and executed experiments and wrote an initial version of the manuscript. BO constructed the SAGE libraries, with assistance from OCG and RC. GV, BO and GRA identified candidate molecules from the SAGE libraries and performed follow-up studies on these molecules. KW, OFE and SWH isolated and tested the CAF/NPF primary cultures. AAT conceived the study, supervised the work and wrote the manuscript. All authors read and approved the manuscript.

## Additional data files

The following additional data are available with the online version of this paper. Additional data file 1 contains the list of transcripts exhibiting a statistically significant difference between the VMP and VSU libraries. Additional data file 2 is the tab delimited text file of the VMP SAGE library. Additional data file 3 is the tab delimited text file of the VSU SAGE library.

## Supplementary Material

Additional data file 1 Presented is a list of transcripts exhibiting a statistically significant difference between the VMP and VSU libraries. Click here for file

Additional data file 2 Presented is the tab delimited text file of the VMP SAGE library. Click here for file

Additional data file 3 Presented is the tab delimited text file of the VSU SAGE library. Click here for file
